# Association of Common Genetic Variants with Lipid Traits in the Indian Population

**DOI:** 10.1371/journal.pone.0101688

**Published:** 2014-07-03

**Authors:** Gagandeep Kaur Walia, Vipin Gupta, Aastha Aggarwal, Mohammad Asghar, Frank Dudbridge, Nicholas Timpson, Nongmaithem Suraj Singh, M. Ravi Kumar, Sanjay Kinra, Dorairaj Prabhakaran, K. Srinath Reddy, Giriraj Ratan Chandak, George Davey Smith, Shah Ebrahim

**Affiliations:** 1 South Asia Network for Chronic Disease (SANCD), Public Health Foundation of India (PHFI), New Delhi, India; 2 Department of Anthropology, University of Delhi, Delhi, India; 3 Department of Anthropology, Rajiv Gandhi University, Itanagar, Arunachal Pradesh, India; 4 Department of Non-Communicable Disease Epidemiology, London School of Hygiene and Tropical Medicine, London, United Kingdom; 5 School of Social and Community Medicine, University of Bristol, Bristol, United Kingdom; 6 Centre for Cellular and Molecular Biology, Hyderabad, Telangana, India; 7 Centre for Chronic Disease Control, New Delhi, India; 8 Public Health Foundation of India, New Delhi, India; National Cancer Institute, National Institutes of Health, United States of America

## Abstract

Genome-wide association studies (GWAS) have been instrumental in identifying novel genetic variants associated with altered plasma lipid levels. However, these quantitative trait loci have not been tested in the Indian population, where there is a poorly understood and growing burden of cardiometabolic disorders. We present the association of six single nucleotide polymorphisms in 1671 sib pairs (3342 subjects) with four lipid traits: total cholesterol, triglycerides, high density lipoprotein cholesterol (HDL-C) and low density lipoprotein cholesterol (LDL-C). We also investigated the interaction effects of gender, location, fat intake and physical activity. Each copy of the risk allele of rs964184 at *APOA1* was associated with 1.06 mmol/l increase in triglycerides (SE = 0.049; p = 0.006), rs3764261 at *CETP* with 1.02 mmol/l increase in both total cholesterol (SE = 0.042; p = 0.017) and HDL-C (SE = 0.041; p = 0.008), rs646776 at *CELSR2-PSRC1-*SORT1 with 0.96 mmol/l decrease in cholesterol (SE = 0.043; p = 0.0003) and 0.15 mmol/l decrease in LDL-C levels (SE = 0.043; p = 0.0003) and rs2954029 at *TRIB1* with 1.02 mmol/l increase in HDL-C (SE = 0.039; p = 0.047). A combined risk score of *APOA1* and *CETP* loci predicted an increase of 1.25 mmol/l in HDL-C level (SE = 0.312; p = 0.0007). Urban location and sex had strong interaction effects on the genetic association of most of the studied loci with lipid traits. To conclude, we validated four genetic variants (identified by GWAS in western populations) associated with lipid traits in the Indian population. The interaction effects found here may explain the sex-specific differences in lipid levels and their heritability. Urbanization appears to influence the nature of the association with GWAS lipid loci in this population. However, these findings will require replication in other Indian populations.

## Introduction

Coronary heart disease is projected to be the leading cause of death for adult Indians by 2020 [1] due to rising prevalence of cardiometabolic disorders [2], [3]. Plasma lipid concentrations are established risk factors for coronary artery disease (CAD) [4] and are also targets for therapeutic interventions [5]. While genome-wide association studies (GWAS) have been instrumental in identifying the quantitative trait loci (QTL) associated with altered levels of plasma lipids [6]–[9], these new discoveries require validation in different population groups in order to understand their wider potential for application and clinical benefits.

Only two previous validations of a limited sub-set of GWAS lipid findings have been reported for Indian populations [10], [11]. During the discovery and replication phases, samples from the LOLIPOP cohort have been widely used to validate GWAS loci for Indian populations, but this cohort comprises Indians residing in the UK and demonstrated a replication rate of 35% [8]. Further, considerable Asian/European differences in lipid profiles have been reported for Asian Indians exhibiting an adverse lipid pattern consisting of low high density lipoprotein cholesterol (HDL-C) and high triglycerides irrespective of diabetic status [12]. Moreover, none of the published reports addressed the complexity of numerous endogamous groups where the average allele frequency differentiation across different groups is known to be 3-fold greater than that observed in European population groups [13]. This indicates a gap in the understanding of the aetiology of lipid traits in Indian populations.

In addition to plasma lipids, other risk factors (e.g. obesity, diabetes and hypertension) are independently and interactively associated with increased risk of cardiovascular diseases [14]–[16] which are further associated with dyslipidemia [17]. Gottesman and colleagues [18] investigated the overlap of genetic variants related to cardiometabolic traits and reported 44 positional genes that have pleiotropic effects. With these findings in mind, we hypothesize that dyslipidemia and metabolic phenotypes such as hyperglycemia, hypertension and anthropometric traits have a common genetic basis.

In our previous study, we had reported on association analysis of five lipid-related QTLs in the Indian population [10]. Since our earlier report, a genome-wide meta-analysis [8] has reported 95 loci associated with lipid levels with an impact in three non-European populations including South Asians. Simultaneously, a non coding genetic variant in the *SORT1* gene was observed that lead to clinical phenotypes, thus suggesting a novel regulatory pathway [9]. Further, a genome-wide meta-analysis found five new loci associated with CAD in European and South Asian populations [19]. In the present study, we raise the following questions: (i) are lipid-related QTLs discovered since our earlier study also associated with altered plasma lipid levels in Indian populations? and (ii) are these genetic loci associated with other cardiometabolic traits in Indians? Answering these questions will help in determining whether cardiometabolic traits have a common pathophysiology across different population groups.

## Methodology

### Ethics statement

The ethical approval for the Indian Migration Study (IMS) was attained from All India Institute of Medical Sciences (AIIMS), New Delhi, India (reference number A-60/4/8/2004). Pre-informed written consent was obtained from each participant before beginning the data collection.

### Study population

The present study was carried out using trait data and DNA from the IMS where migrant and non-migrant factory workers and their co-resident spouses were recruited along with their rural-dwelling sibs [20], [21]. The fieldwork for the IMS took place from 2005–2007 in four factories located in different cities of India (Lucknow, Nagpur, Hyderabad and Bangalore).

### Data collection

Phenotyping details are described in File S1. Briefly, blood pressure, height, weight, waist and hip girth and skin folds were measured on the sib-pairs in the same clinic by trained clinicians and the % body fat was derived from the skin folds. Data on diet and physical activity were recorded on interviewer-administered questionnaires. Fasting blood samples were collected from the participants and the time of the last meal was recorded. Serum and plasma samples were used for generating data on glycemic and lipid profile.

### Genotyping and quality control

Genotyping was performed during 2011–2012 using the Fluidigm platform with single-plex 96.96 chips wherein 96 established GWAS single nucleotide polymorphisms (SNPs) related to cardiometabolic traits were analyzed. Two pairs of duplicates and negative controls (water) were run with every 96 samples for quality control purposes. The genotyping success rate was >95% and duplicate samples had >99% concordance. Out of 96 SNPs, fourteen loci were selected from three major studies on lipid levels [8], [9] and CAD [19]. The limited loci were selected from these studies based on their biological importance and p-values (≤1×10^−40^ for lipid loci and <1×10^−8^ for CAD loci). Out of the 14 SNPs genotyped, nine passed the quality control during data cleaning process and finally six loci were found to be in Hardy-Weinberg equilibrium (HWE) (Table S1 in File S1) for which the results are presented.

### Sample Size and power calculation

We analyzed 1671 sib pairs (3342 individuals) after excluding: (i) singletons (ii) cousin/friend pairs (iii) pairs with one or both sibs having missing phenotypes (iv) pairs with one or both sibs having missing genotyping data on >7 SNPs (v) pairs where one or both sibs self-reported cardiovascular diseases to avoid phenotypic heterogeneity that could cause distorted relationships with lipid traits. Power estimates were derived using the genetic power calculator using option “QTL association for sibships and singletons” [22]. Given the minor allele frequency (MAF) of 21% (minimum MAF in IMS) and the sample size of 1671 sib-pairs, this study had 80% power at α = 0.05 to detect a QTL explaining 1% variation of a trait. Sex-specific associations were estimated among 632 male and 364 female sib-pairs.

### Statistical analysis

After log transformation of skewly distributed variables (see File S1), the association analysis was done using an orthogonal family-based model described by Fulker et al. [23] assuming an additive model of inheritance and considering a sib-pair as the unit of analysis (described in File S1). We applied multi-level models adjusted for age, sex, site (i.e. city) and location (i.e. rural/urban) for analyses on all quantitative traits because these covariates were associated with various outcomes in the study population and differences were found across the sites and locations [20]. Since physical activity and fat intake are important determinants of the lipid profile [24]–[26], we also adjusted for these two variables when estimating the associations. Association of the six selected loci was estimated for four lipid traits [total cholesterol, triglycerides, HDL-C and low density lipoprotein cholesterol (LDL-C)] and also for other metabolic traits related to obesity [body mass index (BMI), waist-hip ratio (WHR), waist circumference (WC) and %body fat], hypertension [systolic blood pressure (SBP) and diastolic blood pressure (DBP)] and diabetes (fasting glucose and fasting insulin) after adjusting for lipid traits and also for WHR in the case of BMI, to detect the independent associations. Correction for multiple testing was not applied for lipid traits as the studied SNPs are established loci [8], [9], [19], whereas for all other metabolic traits inferences were made on the basis of corrected α (value = 0.0083) based on a Bonferroni correction [27] for six tests.

Sex-specific associations were also examined given prior evidence for dimorphic patterns of association [8], [28]. We also tested for interaction effects by sex, location, fat intake and physical activity by including interaction terms within the fixed effect component of the Fulker association model (see details in File S1). Stratified analysis by location, fat intake and physical activity could not be performed due to limited sample size available in these groups.

To estimate the combined effect of loci on lipid levels, risk scores were calculated using loci associated with each of the lipid traits examined in the present study. Weighted risk scores (trait specific β coefficients as weights) based on associated loci observed [29] were fitted into the Fulker model for estimating within sib-pair effects. Since additional samples for estimating the effect of risk score were not available, the present data set was divided into two random halves representing the discovery and validation samples to validate the weighted risk scores.

## Results and Discussion

Over 100 SNPs associated with altered plasma lipid levels have been discovered using GWAS [6]–[9]. Considering that these studies were mostly conducted in populations of European descent and that the minor alleles and their frequency, haplotype background and environmental influences vary across ethnic groups [30], we investigated the role of these loci on four lipid and other traits that predict cardiovascular disease risk in Indian population. Validation of the effects of GWAS loci will likely be more valuable in populations such as Asian Indians [31] that have high disease burden and where conducting GWAS is a difficult task. Table S2 in File S1 shows the comparison between the effect alleles and their frequency observed in European populations and that observed in our study samples and highlights the considerable variation between them. However, the allele frequencies we observed were consistent with those reported for Gujarati Indians living in the Houston (GIH) HapMap database.

The general characteristics of the study population and outcome variables are summarized in Table 1. Significant differences were found between males and females for various cardiometabolic traits, except for total cholesterol and fasting glucose (Table 1).

**Table 1:** Characteristics of sib pairs in Indian Migration Study (N = 1671 pairs).

Characteristic	Total	Males	Females	P
Total Number	3342	1939	1403	
Age (in years)	39.99±10.28	40.79±10.55	38.89±9.80	**<0.001**
Male (%)	58.02	–	–	
Total cholesterol (mmol/l)	4.70±1.13	4.67±1.12	4.74±1.14	0.05
Triglycerides (mmol/l)	1.42±0.70	1.47±0.74	1.35±0.64	**<0.001**
High Density Lipoprotein- Cholesterol (mmol/l)	1.18±0.25	1.16±0.25	1.21±0.25	**<0.001**
Low Density Lipoprotein- Cholesterol (mmol/l)	2.87±0.99	2.83±0.98	2.92±1.01	**0.008**
Systolic Blood Pressure (mmHg)	120.99±16.54	123.42±16.46	117.62±16.05	**<0.001**
Diastolic Blood Pressure (mmHg)	77.01±10.71	77.78±10.92	75.94±10.32	**<0.001**
Fasting Glucose (mmol/l)	5.32±1.41	5.35±1.39	5.28±1.43	0.14
Fasting Insulin (mU/l)	7.53±7.96	7.29±7.93	7.85±8.00	**0.045**
Body mass Index (Kg/m^2^)	23.64±4.48	23.03±3.95	24.49±5.01	**<0.001**
Waist-Hip Ratio	0.87±0.08	0.91±0.07	0.82±0.07	**<0.001**
Waist Circumference (cm)	81.84±11.98	84.45±11.81	78.23±11.25	**<0.001**
% Body Fat	26.64±8.25	23.51±7.34	31.04±7.40	**<0.001**
Average Daily Fat Intake (g/day)	83.62±35.48	89.54±37.59	75.45±30.52	**<0.001**
Total Physical Activity per day (MET hrs/day)	39.00±4.67	39.85±4.99	37.81±3.87	**<0.001**

All values are Mean ± SD; P represents p values on comparison of males and females by T-test.

### Association of six loci with lipid levels

In an earlier report, rs662799 at *APOA5,* rs10503669 at *LPL,* rs780094 in *GCKR*, rs562338 in *APOB* and rs4775041 in *LIPC* were validated in the present study population^10^. In the current analyses, we found associations between genetic variants on/near four loci (*APOA1, CETP, CELSR2-PSRC1-SORT1* and *TRIB1)* and the four lipid traits in the Indian population (Table 2). Although the directions of associations were consistent with that reported worldwide, the effect sizes in the Indian population were larger than that observed for European populations but consistent with other Asian populations (Table S3 in File S1). Of these, rs964184 at *APOA1* locus was associated with 1.06 mmol/l higher triglycerides (SE = 0.049; p = 0.006); rs3764261 at *CETP* with 1.02 mmol/l higher total cholesterol (SE = 0.042; p = 0.017) and 1.02 mmol/l higher HDL-C (SE = 0.041; p = 0.008); rs646776 at *CELSR2-PSRC1-SORT1* with 0.96 mmol/l lower total cholesterol (SE = 0.043; p = 0.0003) and 0.15 mmol/l lower LDL-C (SE = 0.043; p = 0.0003) and rs2954029 at *TRIB1* with 1.02 mmol/l higher HDL-C (SE = 0.039; p = 0.047) levels.

**Table 2:** Within sib-pair association estimates for lipid traits.

			TG			TC			HDL-C			LDL-C		
SNP	Locus	Effect Allele	1β	2SE	3p	1β	2SE	3p	1β	2SE	3p	1β	2SE	3p
rs964184	*APOA1*	G	**0.133**	**0.049**	**0.006**	0.025	0.047	0.596	−0.017	0.046	0.707	−0.006	0.047	0.901
rs3764261	*CETP*	A	0.052	0.043	0.230	**0.099**	**0.042**	**0.017**	**0.108**	**0.041**	**0.008**	0.065	0.042	0.121
rs646776	*CELSR2-PSRC1-SORT1*	G	−0.034	0.045	0.446	−**0.155**	**0.043**	**0.0003**	−0.006	0.042	0.891	−**0.156**	**0.043**	**0.0003**
rs1412444	*LIPA*	C	−0.030	0.041	0.457	0.003	0.039	0.936	0.026	0.038	0.502	0.001	0.039	0.987
rs974819	*PDGFD*	T	0.040	0.042	0.341	−0.012	0.040	0.756	−0.047	0.039	0.232	−0.030	0.040	0.449
rs2954029	*TRIB1*	T	0.029	0.041	0.490	0.049	0.040	0.223	**0.078**	**0.039**	**0.047**	0.034	0.040	0.405

1 β (Z score): within sib-pair coefficient of regression adjusted for age, sex, site (city) and location (rural/urban).

2 SE: Standard Error.

3 Loci were considered to be significant at α = 0.05. TG: trigylcerides; TC: total cholesterol; HDL-C: high density lipoprotein cholesterol; LDL-C: low density lipoprotein cholesterol.

Apolipoprotein A-1 is the major protein component of HDL and promotes cholesterol efflux from tissues to the liver for excretion. The *APOA1* locus was reported to be associated with increased triglycerides and lower HDL-C in the discovery phase of various studies [32]. In subsequent GWAS and meta-analyses, the *APOA1* locus was confirmed to be associated with higher triglycerides, total cholesterol, LDL-C and lower HDL-C levels in Europeans [8]. The association of *APOA1* variants with higher triglyceride levels has also been established in Tibetans [33] as well as in Punjabi and US cohorts [11]. We have also observed significant association of this locus with higher triglyceride levels in the present analyses.

The *CETP* locus codes for cholesteryl ester transfer protein that facilitates the transfer of cholesteryl esters and triglycerides between lipoproteins. *CETP* was found to be associated with high HDL-C in GWAS discovery [34], which was further replicated among Europeans [8], Americans [11] and Punjabi cohorts [11], [35] and with higher total cholesterol levels among Caucasians [8]. Lower triglycerides and LDL-C in a European GWAS meta-analysis were also observed to be associated with *CETP* [8]. In the present study, we validated its association with higher total cholesterol and HDL-C levels.

The third locus is mapped near the *CELSR2-PSRC1-SORT1* gene cluster and emerged from a GWAS of LDL-C conducted among British population [36]. Its association with lower LDL-C levels was also replicated in Austrians [37] and Pakistanis [38]; and with high total cholesterol in Netherland population [39]. In the present study, *CELSR2-PSRC1-SORT1* was associated with lower levels of total cholesterol and LDL-C.

The *TRIB1* locus codes for tribbles homologue 1 protein that regulates the activation of mitogen activated protein kinases. The association of this locus was first reported to be associated with triglycerides [40] and subsequently with low total cholesterol, LDL-C and high HDL-C in European population [8], [41]. Here, we observed its association with higher HDL-C levels, which is in agreement with that seen for Europeans. In contrast, the *TRIB1* locus was associated with lower HDL-C levels in a Danish population [42].

Since lifestyle factors, especially diet and physical activity, are strongly associated with individual serum lipid profiles [24]–[26], dietary daily fat intake and physical activity (total MET score) were included as additional covariates to explore the possible associations of studied QTLs. In the studied population, these adjusted analyses did not alter the direction or effect size compared with the unadjusted analyses of these two covariates (Table S4 in File S1).

The cumulative effect of genetic variants for lipids is known to be associated with subclinical and clinical cardiovascular outcomes [43]. In the present study, multiple loci were associated with HDL-C and total cholesterol, but the directions of the effects were same only for SNPs associated with HDL-C (Table 2). Thus, an attempt was made to estimate the combined effect of the two significant loci (rs2954029 at *TRIB1* and rs3764261 at *CETP*) on HDL-C levels. The weighted risk score was associated with a 1.25 mmol/l higher HDL-C level per risk alleles at both variants (SE = 0.312; p = 0.0007) as opposed to a 1.02 mmol/l increase that could be explained by independent SNPs.

### Association of six loci with related metabolic traits

We further investigated these GWAS loci related to lipids for their association(s) with other metabolic traits which would help in identifying the causal pathways that are common to these outcomes. While there are sufficient epidemiological and clinical evidence that support the relationship among dyslipidemia, cardiovascular disease, diabetes, obesity and hypertension; the common genetic mechanisms underlying these diseases are not well established [18]. Evidence of weak associations between lipid related genetic variants in *LPL* and *GCKR* have been reported earlier with hypertension and variants in *LPL* with fasting glucose, fasting insulin and systolic blood pressure [10]. In the present study, three out of the six investigated loci were associated with metabolic disorders (Tables S5–S7 in File S1). While performing association analyses, adjustments were made for lipid traits (in addition to age, sex, site and location) to avoid bias that could occur due to phenotypic heterogeneity.

Of interest was that the two loci *APOA1* and *TRIB1* that affected HDL-C levels also influenced waist circumference. We noted an overlapping association between lipid levels and waist circumference, which would point towards a common pathophysiology between lipids and obesity traits. In addition, we found a weak association between the *PDGFD* locus and diastolic blood pressure, which echoes the pattern of association with other traits in a previous study [18] that found that *PDGFD* was implicated in variety of functions, especially angiogenesis. Recently, Schierer and colleagues [35] in a similar attempt reported that *CETP* was associated with a decrease in systolic blood pressure (β = −0.08, p = 0.002) among Asian normoglycemic controls.

However, these loci need to be assessed in a larger set of samples in order to draw more meaningful inferences, as none of the genetic variants retained the association after correction for multiple testing.

### Sex-specific association of six loci related to lipid levels

There is evidence that point towards sex heterogeneity in the association of lipid-related loci with lipid parameters [8]. We found sex-specific associations with various lipid traits (Table 3). Out of the four loci that were associated in the combined analyses, *CETP* was associated with 1.05 mmol/l higher HDL-C (SE = 0.071; p = 0.001) and *CELSR2-PSRC1-SORT1* was associated with 0.94 mmol/l lower triglycerides (SE = 0.081; p = 0.074), 0.95 mmol/l lower total cholesterol (SE = 0.076; p = 0.007) and 0.15 mmol/l lower LDL-C (SE = 0.072; p = 0.033) among male sib-pairs only. On the other hand, *APOPA1* was associated with 1.05 mmol/l higher triglycerides (SE = 0.089; p = 0.015) and *TRIB1* with 1.04 mmol/l higher HDL-C (SE = 0.077; p = 0.007) among female sib-pairs only. In addition, *LIPA* was associated with 1.03 mmol/l higher HDL-C (SE = 0.078; p = 0.041) only in female sib-pairs which did not emerge in the combined analyses. We previously also reported sex-specific associations for lipid traits [10] and now postulate that these findings might explain the sex differences in lipid levels and their heritability.

**Table 3:** Within sib-pair association estimates for lipid traits stratified by gender.

SNP	Locus	Effect Allele	Trait	Male Pairs N = 632 1β(2SE, 3p)	Female Pairs N = 364 1β(2SE, 3p)	Interaction_sex_−1 4β (2SE, 3p)	Interaction_sex_−2 5β (2SE, 3p)
rs964184	*APOA1*	G	TG	0.098 (0.085, 0.251)	**0.217 (0.089, 0.015)**	**0.180 (0.051, 0.0004)**	**0.168 (0.051, 0.001)**
			TC	0.056 (0.082, 0.491)	0.000 (0.094, 0.999)	0.018 (0.049, 0.709)	0.009 (0.049, 0.863)
			HDL-C	0.000 (0.080, 0.999)	**−**0.104 (0.093, 0.262)	**−**0.077 (0.049, 0.114)	**−**0.079 (0.049, 0.107)
			LDL-C	0.050 (0.079, 0.530)	**−**0.035 (0.095, 0.708)	**−**0.011 (0.049, 0.825)	**−**0.018 (0.050, 0.714)
rs3764261	*CETP*	A	TG	0.112 (0.080, 0.160)	**−**0.066 (0.076, 0.386)	**0.129 (0.042, 0.002)**	**0.121 (0.043, 0.004)**
			TC	0.109 (0.076, 0.151)	0.086 (0.080, 0.284)	0.043 (0.041, 0.292)	0.034 (0.041, 0.409)
			HDL-C	**0.247 (0.071, 0.001)**	**−**0.003 (0.079, 0.972)	0.000 (0.040, 0.996)	0.004 (0.041, 0.918)
			LDL-C	0.024 (0.073, 0.740)	0.108 (0.081, 0.183)	0.009 (0.041, 0.822)	**−**0.001 (0.042, 0.979)
rs646776	*CELSR2-PSRC1-SORT1*	G	TG	**−0.144 (0.081, 0.074)**	0.123 (0.083, 0.138)	**−**0.001 (0.047, 0.986)	**−**0.006 (0.048, 0.906)
			TC	**−0.205 (0.076, 0.007)**	**−**0.016 (0.087, 0.857)	**−0.126 (0.045, 0.005)**	**−0.135 (0.045, 0.003)**
			HDL-C	**−**0.056 (0.075, 0.454)	0.067 (0.085, 0.429)	**−0.115 (0.045, 0.011)**	**−0.123 (0.046, 0.007)**
			LDL-C	**−0.154 (0.072, 0.033)**	**−**0.095 (0.087, 0.273)	**−0.093 (0.045, 0.040)**	**−0.099 (0.046, 0.030)**
rs1412444	*LIPA*	C	TG	**−**0.062 (0.072, 0.389)	0.038 (0.076, 0.621)	0.055 (0.034, 0.103)	0.043 (0.034, 0.207)
			TC	0.007 (0.068, 0.917)	0.010 (0.080, 0.901)	**−**0.041 (0.033, 0.206)	**−**0.055 (0.033, 0.096)
			HDL-C	0.010 (0.066, 0.882)	**0.159 (0.078, 0.041)**	**−0.092 (0.032, 0.004)**	**−0.100 (0.033, 0.002)**
			LDL-C	0.022 (0.065, 0.731)	**−**0.066 (0.081, 0.413)	**−**0.044 (0.033, 0.186)	**−**0.055 (0.033, 0.098)
rs974819	*PDGFD*	T	TG	0.023 (0.071, 0.752)	**−**0.014 (0.072, 0.849)	**0.093 (0.040, 0.020)**	**0.079 (0.040, 0.050)**
			TC	0.019 (0.067, 0.779)	0.031 (0.076, 0.678)	**−**0.018 (0.038, 0.639)	**−**0.031 (0.039, 0.421)
			HDL-C	**−**0.120 (0.065, 0.066)	**−**0.065 (0.074, 0.376)	**−0.124 (0.038, 0.001)**	**−0.124 (0.038, 0.001)**
			LDL-C	0.019 (0.064, 0.770)	0.046 (0.076, 0.544)	**−**0.031 (0.039, 0.425)	**−**0.042 (0.039, 0.281)
rs2954029	*TRIB1*	T	TG	0.106 (0.071, 0.136)	**−**0.039 (0.076, 0.602)	**0.114 (0.039, 0.003)**	**0.107 (0.039, 0.007)**
			TC	0.109 (0.067, 0.103)	0.063 (0.080, 0.431)	0.028 (0.037, 0.456)	0.025 (0.038, 0.508)
			HDL-C	0.050 (0.066, 0.447)	**0.207 (0.077, 0.007)**	**−**0.057 (0.037, 0.127)	**−**0.057 (0.038, 0.129)
			LDL-C	0.091 (0.065, 0.158)	0.034 (0.081, 0.673)	0.031 (0.038, 0.408)	0.0209 (0.038, 0.454)

1 β (Z score): within sib-pair coefficient of regression adjusted for age, site (city) and location (rural/urban).

2 SE: Standard Error.

3 Loci were considered to be significant at α = 0.05.

4 β (Z score): within sib-pair interaction coefficient of regression adjusted for age, site (city) and location (rural/urban).

5 β (Z score): within sib-pair interaction coefficient of regression adjusted for age, site (city), location (rural/urban), fat intake and physical activity (total MET score) TG: trigylcerides; TC: total cholesterol; HDL-C: high density lipoprotein cholesterol; LDL-C: low density lipoprotein cholesterol.

The exploratory interaction analyses provide evidence that the genetic effects of all six loci were influenced by gender and these associations were consistent even after adjustments for fat intake and physical activity (Table 3). Modifications in the genetic effects of two loci was seen where the effects were stronger among males in the case of *APOA1* with triglycerides (β = 0.168, SE = 0.051, p = 0.001) and *CELSR2-PSRC1-SORT1I* with total cholesterol (β = −0.135, SE = 0.045, p = 0.003) and LDL-C (β = −0.099, SE = 0.046, p = 0.030). In addition, a few conditional associations with sex were found, such as association of *LIPA* with HDL-C (β = −0.100, SE = 0.033, p = 0.002) (Table 3) that did not originate in main effects.

### Effects of environmental factors on lipid loci

Rural to urban migration has been suggested to be associated with increased fat intake and reduced physical activity [20]. Thus, we tested for effect modification by location, fat intake and physical activity while allowing for the main effects of four loci that were associated with the lipid traits. Genetic associations of four loci with lipids was found in urban dwellers compared to their rural sibs after adjusting for daily fat intake and physical activity (Table 4), suggesting interaction. The genetic effect of *APOA1* on triglycerides (β = 0.147, SE = 0.044, p = 0.001) and *CETP* on total cholesterol (β = 0.110, SE = 0.035, p = 0.002) increased while interacting with location when compared to the main effects (see Table 2). Further, conditional associations with urban location were found, such as the association of *LIPA* with total cholesterol (β = 0.082, SE = 0.030, p = 0.006) which was not evident in the main effects.

**Table 4:** Within sib-pair interaction estimates for lipid traits by location, average daily fat intake and physical activity.

**SNP**	**Locus**	**Effect Allele**	**Trait**	**Interaction _location_−1** 1 **β (** 2 **SE,** 3 **p)**	**Interaction _location_−2** 4 **β (** 2 **SE,** 3 **p)**	**Interaction_ fat_** 5 **β** **(** 2 **SE,** 3 **p)**	**Interaction_ MET_** 6 **β (** 2 **SE,** 3 **p)**
rs964184	*APOA1*	G	TG	**0.172 (0.044, 0.0001)**	**0.147 (0.044, 0.001)**	**0.107 (0.052, 0.040)**	**−**0.058 (0.051, 0.248)
			TC	**0.092 (0.043, 0.030)**	0.063 (0.043, 0.145)	0.077 (0.051, 0.128)	0.031 (0.049, 0.528)
			HDL-C	0.008 (0.042, 0.847)	0.009 (0.043, 0.836)	**−**0.022 (0.050, 0.655)	0.049 (0.048, 0.312)
			LDL-C	0.048 (0.043, 0.262)	0.021 (0.043, 0.626)	0.057 (0.051, 0.262)	0.030 (0.049, 0.536)
rs3764261	*CETP*	A	TG	**0.116 (0.036, 0.001)**	**0.093 (0.037, 0.012)**	0.050 (0.043, 0.238)	**−**0.016 (0.042, 0.710)
			TC	**0.141 (0.035, 0.0001)**	**0.110 (0.035, 0.002)**	**0.097 (0.041, 0.018)**	0.001 (0.040, 0.990)
			HDL-C	0.053 (0.034, 0.120)	0.048 (0035, 0.169)	0.023 (0.041, 0.569)	0.033 (0.040, 0.403)
			LDL-C	**0.096 (0.035, 0.006)**	0.070 (0.036, 0.051)	**0.085 (0.041, 0.042)**	**−**0.010 (0.041, 0.804)
rs646776	*CELSR2-PSRC1-SORT1*	G	TG	0.023 (0.040, 0.572)	0.004 (0.041, 0.925)	**−**0.032 (0.049, 0.522)	**−**0.076 (0.045, 0.095)
			TC	**−**0.011 (0.039, 0.339)	**−**0.037 (0.039, 0.339)	**−**0.092 (0.047, 0.051)	**−**0.081 (0.043, 0.062)
			HDL-C	**−**0.025 (0.038, 0.509)	**−**0.026 (0.039, 0.507)	**−**0.060 (0.047, 0.201)	**−**0.002 (0.043, 0.961)
			LDL-C	**−**0.007 (0.039, 0.853)	**−**0.033 (0.039, 0.403)	**−**0.061 (0.047, 0.200)	**−**0.063 (0.043, 0.145)
rs1412444	*LIPA*	C	TG	**0.065 (0.030, 0.032)**	0.034 (0.031, 0.278)	**−**0.027 (0.036, 0.455)	**−0.077 (0.033, 0.021)**
			TC	**0.115 (0.029, 0.0001)**	**0.082 (0.030, 0.006)**	0.037 (0.034, 0.276)	**−**0.008 (0.032, 0.804)
			HDL-C	0.022 (0.028 (0.445)	0.014 (0.029, 0.634)	**−**0.015 (0.034, 0.653)	0.032 (0.032, 0.308)
			LDL-C	**0.102 (0.029, 0.0005)**	**0.076 (0.030, 0.011)**	0.056 (0.035, 0.105)	0.000 (0.032, 0.999)
rs974819	*PDGFD*	T	TG	**0.085 (0.035, 0.016)**	0.057 (0.036, 0.113)	**−**0.001 (0.042, 0.983)	**−**0.070 (0.039, 0.075)
			TC	**0.070 (0.034, 0.039)**	0.037 (0.035, 0.283)	0.029 (0.040, 0.472)	**−**0.019 (0.038, 0.625)
			HDL-C	**−**0.039 (0.033, 0.240)	**−**0.047 (0.034, 0.166)	0.007 (0.040, 0.866)	0.005 (0.037, 0.896)
			LDL-C	0.055 (0.034, 0.109)	0.028 (0.035, 0.427)	0.034 (0.041, 0.410)	**−**0.017 (0.038, 0.648)
rs2954029	*TRIB1*	T	TG	0.060 (0.035, 0.088)	0.031 (0.036, 0.390)	0.044 (0.040, 0.269)	**−**0.030 (0.040, 0.449)
			TC	**0.132 (0.034, 0.0001)**	**0.102 (0.034, 0.003)**	**0.114 (0.038, 0.003)**	0.016 (0.038, 0.671)
			HDL-C	0.029 (0.033, 0.392)	0.027 (0.034, 0.443)	0.062 (0.038, 0.105)	**0.087 (0.038, 0.021)**
			LDL-C	**0.126 (0.034, 0.0001)**	**0.103 (0.035, 0.003)**	**0.111 (0.039, 0.004)**	0.012 (0.039, 0.751)

1 β (Z score): within sib-pair interaction coefficient of regression for location (rural/urban) adjusted for age, sex and site (city).

2 SE: Standard Error.

3 Loci were considered to be significant at α = 0.05.

4 β (Z score): within sib-pair interaction coefficient of regression for location (rural/urban) adjusted for age, sex, site (city), fat intake and physical activity (total MET score).

5 β (Z score): within sib-pair interaction coefficient of regression for fat intake adjusted for age, sex, site (city), location (rural/urban) and total MET score**.**

6 β (Z score): within sib-pair interaction coefficient of regression for physical activity (total MET score) adjusted for age, sex, site (city), location (rural/urban) and fat intake. TG: trigylcerides; TC: total cholesterol; HDL-C: high density lipoprotein cholesterol; LDL-C: low density lipoprotein cholesterol.

Similarly, in comparison to the main effects (Table 2), reduction in the genetic effects of *AOPA1* on triglycerides (β = 0.107, SE = 0.052, p = 0.040) and *CETP* on total cholesterol (β = 0.097, SE = 0.041, p = 0.018) were seen in people consuming high dietary fat after adjusting for physical activity (Table 4). Further, conditional associations with dietary fat was seen for *CETP* on LDL-C (β = 0.085, SE = 0.041, p = 0.042) and *TRIB1* on total cholesterol (β = 0.114, SE = 0.038, p = 0.003) and LDL-C (β = 0.111, SE = 0.039, p = 0.004) which were absent in the main effects.

The genetic effect of *TRIB1* on HDL-C (β = 0.087, SE = 0.038; p = 0.021) was found to be stronger among physically active participants (Talbe 4) than the main effects (Table 2) after adjusting for fat intake. A conditional association of *LIPA* on triglycerides (β = −0.077, SE = 0.033; p = 0.021) was also seen among physically active individuals (Table 4).

To conclude, we confirm that four previously discovered QTLs in Europeans also influence lipid levels in the Indian population. Two of these loci (*TRIB1* and *CELSR2-PSRC1-SORT1*) have been validated in the Indian population for the first time. However, the present findings will need to be replicated in larger samples. Sex-specific associations were also observed in the studied population along with strong interaction effects for all six loci studied. Genetic associations with lipid traits were stronger in urban dwellers compared to their rural sibs, suggesting interaction. Some evidence was also seen for interaction by dietary fat intake and physical activity on the genetic association of lipid traits.

## Supporting Information

File S1 **Supporting Information containing details of methodology and Tables S1–S7.**
(DOC)
