# Supplementary material for: Association of Common Genetic Variants with Lipid Traits in the Indian Population
Source: PLoS One. 2014 Jul 3;9(7):e101688. doi: 10.1371/journal.pone.0101688 (PMC4081649; doi:10.1371/journal.pone.0101688)
Supplement: File S1 — Supporting Information containing details of methodology and Tables S1–S7. (DOC) [file pone.0101688.s001.doc]

**Supplementary Information**

**Biochemical Phenotypes:** Fasting blood samples were collected from the participants after having a pre-informed written consent and the time of last meal was recorded. Serum and plasma samples were separated on the same day of collection and stored in -20^o^C at local sites and were then transported to AIIMS, New Delhi on regular basis. Plasma glucose was measured on the same day of sampling in local laboratories of each site with the GOD-PAP method [1] using kits from Randox Laboratory Ltd. (Crumlin City, United Kingdom) and rest of the biochemical assays were performed in the AIIMS coordinating laboratory. Fasting insulin was assayed in serum samples by the ELISA method, as a solid phase two-site enzyme immunoassay, using kits from MERCODIA [2]. Serum high density lipoprotein cholesterol (HDL-C) was estimated directly by an elimination method [3]; total cholesterol was estimated by an enzymatic endpoint method; and triglycerides (TG) by GPO-PAP method [1]. Low density lipoprotein cholesterol (LDL-C) level was estimated using standard Friedewald-Fredrickson formula [4]. The quality of local assays was regularly monitored by AIIMS and was also checked with regular external standards and internal duplicate assays. For quality assurance the Cardiac Biochemistry Lab, AIIMS, is part of the UK National External Quality Assessment (http://www.ukneqas.org.uk/).

**Physiological Measurements:** Blood pressure was measured using an Omron M5-I automatic machine in the sitting position using an appropriate sized cuff on the right upper arm after a period of 5 min rest. Three consecutive readings were taken each for systolic (SBP) and diastolic (DBP) blood pressure with a gap of 1 minute in between and then average of the last two was considered for the analysis.

**Anthropometric Measurements:** Standing height was measured with mandibular stretch at end of expiration using a plastic stadiometer (Leicester height measure, Chasmors Ltd, London). Weight was measured in light clothes with shoes off using a digital scale (Model-PS16, Beurer, Germany). Waist and hip circumferences (WC and HC) were measured using a nonstretch narrow metal tape with a blank lead in (Chasmors metallic tape). The percentage body fat was calculated using standard formulae from triceps and subscapular skinfold measures using Holtain calipers [5]. Height, weight and circumferences were measured twice and skinfolds were measured thrice and mean of the repeated measures was taken for all the traits. The acceptable difference between the readings were less than equal to 0.5cm for height, 0.5kg for weight, 0.5cm for circumferences and 0.1mm for skin folds. Body mass index (BMI) was calculated as weight in kilograms divided by the squared product of height measured in metres while waist-hp ratio (WHR) was calculated by dividing waist girth by hip girth.

**Lifestyle Variables:** Data to assess diet and physical activity were collected by administering questionnaires. Daily fat intake (g/day) was calculated from a food frequency questionnaire, in which participants were asked about average consumption (daily, weekly, monthly or yearly) of 184 different food items consumed in last year [6]. IMS-PAQ questionnaire was administered to gather information on 42 different types of physical activities performed in previous month and thereafter metabolic equivivalent task (MET) unit values were assigned to each activity using the compendium of physical activity and WHO/FAO/UN guidelines [7,8]. The total MET scores (hrs/day) were calculated accounting for the total physical activity performed in a day wherein 1MET is equivalent to energy spent whilst sitting quietly [9].

**Statistical Analysis:** All the continuous variables were checked for normal distribution and log transformations were done for skewed variables and were then transformed into z scores prior to analysis for the interpretation of β in terms of change in SE per allele. LDL-C, WHR, WC and %body fat were not log transformed as they were showing normal distribution and were thus directly z transformed for analysis.

Hardy-Weinberg equilibrium was estimated on urban dwelling unrelated individuals (factory workers and co-resident spouse, N=1713) using exact test significance probability after applying Sidák correction for multiple comparisons [10] which provided corrected α of 0.00568.

Fulker association model is a mixed regression model in which the genetic effect is decomposed into fixed between- and within-family effects, where we modeled shared environmental and residual genetic effects within families (pair variable in the present study) as random effects. Inferences were drawn from within-family effect as it is resistant to population stratification.

In gene-environment interaction analyses, the product of genetic variant score and environmental risk factor score was fitted in the Fulker association model instead of only genetic score to estimate the within sib-pair effects. The genetic scores of 0, 1 and 2 were given based on number of risk alleles while the environmental risk factor of 0 and 1 were given based on presence of that risk factor. Few examples of scorings are provided below:

| ID | *rs54678 | SNP | #Location | Interaction (SNP X Location) |
| --- | --- | --- | --- | --- |
| 1 | CT | 1 | 1 | 1 |
| 2 | CT | 1 | 0 | 0 |
| 3 | TT | 2 | 1 | 2 |
| 4 | CT | 1 | 0 | 0 |
| 5 | CC | 0 | 1 | 0 |

*Risk allele: T; #Location: urban=1, rural=0

While estimating effect of location (rural/urban) by performing interaction analysis, urban location was kept as the exposure and the model was adjusted for age, gender and site (city). The interaction effects by location was also estimated after adjusting for fat intake and physical activity as these two lifestyle variables vary in rural and urban locations. Similarly, while estimating interaction effect by gender, male sex was kept as the exposure and model was adjusted for age, site (city) and location (rural/urban) and then separately for fat intake and physical activity as well. For interaction by daily fat intake and physical activity, the median values of average daily fat intake (76.9838g/day) and average daily total MET score (38.2083 hrs/day) were considered as cut offs and the exposure was defined as having ≥ median value. Thereafter the model was adjusted for age, sex, site (city), location (rural/urban) and total MET score for estimating fat intake interaction while the model was adjusted for age, sex, site (city), location (rural/urban) and fat intake for estimating physical activity interaction.

**References**

1. Trinder P. (1969). Determination of blood glucose using 4-amino phenazone as oxygen acceptor. J Clin Path. 22:246.
2. [Sjöstrand M](http://www.ncbi.nlm.nih.gov/pubmed?term=Sjöstrand M%5BAuthor%5D&cauthor=true&cauthor_uid=15616023), [Gudbjörnsdottir S](http://www.ncbi.nlm.nih.gov/pubmed?term=Gudbjörnsdottir S%5BAuthor%5D&cauthor=true&cauthor_uid=15616023), [Strindberg L](http://www.ncbi.nlm.nih.gov/pubmed?term=Strindberg L%5BAuthor%5D&cauthor=true&cauthor_uid=15616023), [Lönnroth P](http://www.ncbi.nlm.nih.gov/pubmed?term=Lönnroth P%5BAuthor%5D&cauthor=true&cauthor_uid=15616023). (2005) Delayed transcapillary delivery of insulin to muscle interstitial fluid after oral glucose load in obese subjects. Diabetes. 54:152-157
3. Izawa S, Okada M, Matsui H, Horita Y. (1997) A new direct method for measuring HDL-cholesterol which does not produce any biased values. J Med Pharm Sci. 37:1385–1388.
4. Friedewald WT, Levy RI, Fredrickson DS. (1972) Estimation of the concentration of low-density lipoprotein cholesterol in plasma, without use of the preparative ultracentrifuge. Clin Chem. 18:499-502.
5. Durnin JV, Womersley J. (1974) Body fat assessed from total body density and its estimation from skinfold thickness: measurements on 481 men and women aged from 16 to 72 years. Br J Nutr. 32:77-97.
6. Bowen L, Ebrahim S, De Stavola B, Ness A, Kinra S, et al. (2011) Dietary intake and rural-urban migration in India: a cross-sectional study. [PLoS One.](http://www.ncbi.nlm.nih.gov/pubmed?term=A Replication Study of GWAS-Derived Lipid Genes in Asian Indians%3A The Chromosomal Region 11q23.3 Harbors Loci Contributing to Triglycerides) 6:e14822.
7. Ainsworth BE, Haskell WL, Whitt MC, Irwin ML, Swartz AM, et al. (2000) Compendium of physical activities: an update of activity codes and MET intensities. Med Sci Sports Exerc. 32:S498–S504.
8. FAO/WHO/UNU. (2005) Human energy requirements. Scientific background papers from the Joint FAO/WHO/UNU Expert Consultation. Rome, Italy. Pp 35–52.
9. Sullivan R, Kinra S, Ekelund U, Bharathi AV, Mario V, et al. (2012) Evaluation of the Indian migration study physical activity questionnaire (IMS-PAQ): a cross-sectional study. Int j Behav Nutr Phy Act. 9:13
10. Sidák Z. (1976) Rectangular confidence regions for the means of multivariate normal distributions. J Am Stat Assoc. 62:626-633.

**Table S1: Hardy-Weinberg Equilibrium exact test probability values**

| **SNP** | **Locus** | **Effect Allele** | **Lucknow**  **(N=575)** | **Nagpur (N=451)** | **Hyderabad (N=360)** | **Bangalore (N=286)** | **Combined (N=1672)** |
| --- | --- | --- | --- | --- | --- | --- | --- |
| rs2131925 | *ANGPTL3* | G | 0.5490 | 0.0573 | 0.0705 | 0.4040 | **0.0056** |
| rs964184 | *APOA1* | G | 0.1172 | 0.4678 | 0.4560 | 0.1748 | 0.0235 |
| rs3764261 | *CETP* | A | 0.2573 | 0.5948 | 0.3037 | 1.0000 | 0.3331 |
| rs646776 | *CELSR2-PSRC1-SORT1* | G | 0.3294 | 1.0000 | 0.4048 | 0.0661 | 0.3154 |
| rs1412444 | *LIPA* | C | 0.7998 | 0.1544 | 0.4581 | 0.5534 | 0.1260 |
| rs12678919 | *LPL* | G | **0.0008** | **0.0003** | 0.2281 | 0.4847 | **0.0001** |
| rs974819 | *PDGFD* | T | 0.4506 | 0.9175 | 0.7173 | 1.0000 | 0.5101 |
| rs12740374 | *SORT1* | T | **0.0023** | 0.5033 | 0.7660 | **0.0006** | **0.0001** |
| rs2954029 | *TRIB1* | T | 0.7108 | 0.4850 | 1.0000 | 0.5951 | 0.3879 |

Note: Loci were considered to be significant or in HW disequilibrium at α=0.0057 after SIDAK correction for multiple testing

**Table S2: Allelic distribution at various single nucleotide polymorphisms (SNPs) genotyped in the present study and comparison with the published data**

| **^1^SNP** | **^2^Locus** | **^3^Allele** | **^4^Allele frequency in Europeans** | **^5^Reference** | **^6^Allele frequency in IMS** | **^7^ Allele frequency in GIH** |
| --- | --- | --- | --- | --- | --- | --- |
| rs964184 | *APOA1* | G | 0.13 | [8] | 0.21 | 0.19 |
| rs3764261 | *CETP* | A | 0.32 | [8] | 0.33 | 0.39 |
| rs646776 | *CELSR2-PSRC1-SORT1* | G | 0.21 | [38] | 0.27 | 0.27 |
| rs1412444 | *LIPA* | C | 0.66 | [19] | 0.47 | 0.41 |
| rs974819 | *PDGFD* | T | 0.29 | [19] | 0.34 | 0.31 |
| rs2954029 | *TRIB1* | T | 0.47 | [8] | 0.35 | -- |

^1^SNP:single nucleotide polymorphism ( GWAS SNPs selected for present study). ^2^Locus represent either a plausible biological candidate gene at the locus or the nearest annotated gene to the SNP. ^3^Effect allele (minor allele) examined in present study. ^4^Allele frequency observed in European populations. ^5^Reference article from where the information on the SNP was gathered. ^6^Allele frequency observed in present study (IMS) samples. ^7^Allele frequency reported in the HapMap database for Gujratai Indians in Houston (GIH) population.

Table S3: Comparison of effect sizes of significant SNPs on lipid traits as observed in IMS and comparison with other studies

| **SNP** | **Locus** | **Effect Allele** | **Trait** | **^1^β (SE)** | **^2^Effect**  **(mmol/l)** | **Population / Study** | **Reference** |
| --- | --- | --- | --- | --- | --- | --- | --- |
| rs964184 | *APOA1* | G | TG | 0.133 (0.049)  0.15  --  --  --  0.18 | 1.06  --  1.4  0.19  0.003  -- | Indian (Present Study)  Indian (Punjabi)  Tibetan  Caucasian  Framingham Heart Study  American | --  [11]  [35]  [8]  [34]  [11] |
| rs3764261 | *CETP* | A | TC | 0.099 (0.042)  -- | 1.02  0.04 | Indian (Present Study)  Caucasian | --  [8] |
|  |  |  | HDL-C | 0.108 (0.041)  0.09  --  --  0.10 | 1.02  --  0.09  0.09  -- | Indian (Present Study)  Indian (Punjabi)  FUSION cohort (Finland)  Caucasian  American | --  [11]  [36]  [8]  [11] |
| rs646776 | *CELSR2-PSRC1-SORT1* | G | TC | -0.155 (0.043)  -- | -0.96  0.17 | Indian (Present Study)  Netherland | --  [41] |
|  |  |  | LDL-C | -0.156 (0.0003)  --  -0.16 (0.03)  -3.18 (0.92) | -0.15  -0.16  --  -- | Indian (Present Study)  Pakistani  UK Cohort  Caucasians | --  [40]  [38]  [39] |
| rs2954029 | *TRIB1* | T | HDL-C | 0.078 (0.039)  --  --  0.012 (0.003) | 1.02  0.02  -0.04  -- | Indian (Present Study)  Caucasian  Dannish  8 cohorts | --  [8]  [33]  [32] |

TG: trigylcerides; TC: total cholesterol; HDL-C: high density lipoprotein cholesterol; LDL-C: low density lipoprotein cholesterol.

^1^Effect sizes in terms of standardized z scores. ^2^Raw effect sizes calculated after back transformations.

**Table S4: Within sib-pair association estimates for lipid traits (including physical activit**y and fat intake as covariates)

|  |  |  | **TG** | | | **TC** | | | **HDL-C** | | | **LDL-C** | | |
| --- | --- | --- | --- | --- | --- | --- | --- | --- | --- | --- | --- | --- | --- | --- |
| **SNP** | **Locus** | **Effect Allele** | **^1^β** | **^2^SE** | **^3^p** | **^1^β** | **^2^SE** | **^3^p** | **^1^β** | **^2^SE** | **^3^p** | **^1^β** | **^2^SE** | **^3^p** |
| rs964184 | *APOA1* | G | 0.128 | 0.049 | **0.009** | 0.029 | 0.047 | 0.539 | -0.010 | 0.047 | 0.828 | -0.004 | 0.048 | 0.928 |
| rs3764261 | *CETP* | A | 0.057 | 0.043 | 0.186 | 0.100 | 0.042 | **0.016** | 0.102 | 0.041 | **0.013** | 0.069 | 0.042 | 0.102 |
| rs646776 | *CELSR2-PSRC1-SORT1* | G | -0.026 | 0.045 | 0.558 | -0.153 | 0.043 | **0.0003** | -0.010 | 0.043 | 0.814 | -0.156 | 0.043 | **0.0003** |
| rs1412444 | *LIPA* | C | -0.035 | 0.041 | 0.397 | -0.001 | 0.039 | 0.979 | 0.016 | 0.039 | 0.676 | -0.001 | 0.040 | 0.984 |
| rs974819 | *PDGFD* | T | 0.039 | 0.042 | 0.355 | -0.015 | 0.040 | 0.703 | -0.047 | 0.039 | 0.235 | -0.032 | 0.040 | 0.428 |
| rs2954029 | *TRIB1* | T | 0.032 | 0.042 | 0.440 | 0.060 | 0.040 | 0.133 | 0.078 | 0.040 | **0.049** | 0.046 | 0.040 | 0.256 |

^1^β (Z score): within sib-pair coefficient of regression adjusted for age, sex, site (city), location (rural/urban), total MET Score (physical activity) and fat intake ^2^SE: Standard Error. ^3^Loci were considered to be significant at α=0.05 TG: trigylcerides; TC: total cholesterol; HDL-C: high density lipoprotein cholesterol; LDL-C: low density lipoprotein cholesterol

**Table S5: Within sib-pair association estimates for** blood pressure

|  |  |  | **Systolic Blood Pressure** | | | **Diastolic Blood Pressure** | | |
| --- | --- | --- | --- | --- | --- | --- | --- | --- |
| **SNP** | **Locus** | **Effect Allele** | **^1^β** | **^2^SE** | **^3^p** | **^1^β** | **^2^SE** | **^3^p** |
| rs964184 | *APOA1* | G | -0.040 | 0.045 | 0.370 | -0.071 | 0.046 | 0.124 |
| rs3764261 | *CETP* | A | 0.024 | 0.040 | 0.552 | -0.003 | 0.041 | 0.943 |
| rs646776 | *CELSR2-PSRC1-SORT1* | G | -0.021 | 0.041 | 0.600 | -0.011 | 0.042 | 0.790 |
| rs1412444 | *LIPA* | C | 0.015 | 0.037 | 0.686 | -0.002 | 0.038 | 0.959 |
| rs974819 | *PDGFD* | T | 0.058 | 0.038 | 0.125 | 0.080 | 0.039 | **0.041** |
| rs2954029 | *TRIB1* | T | -0.066 | 0.038 | 0.082 | -0.022 | 0.039 | 0.571 |

^1^β (Z score): within sib-pair coefficient of regression adjusted for age, sex, site (city) and location (rural/urban) and lipid traits ^2^SE: Standard Error. ^3^Loci were considered to be significant at α=0.0083 after false discovery rate for multiple testing

**Table S6: Within sib-pair association estimates for various g**lycemic traits

|  |  |  | **Fasting Glucose** | | | **Fasting Insulin** | | |
| --- | --- | --- | --- | --- | --- | --- | --- | --- |
| **SNP** | **Locus** | **Effect Allele** | **^1^β** | **^2^SE** | **^3^p** | **^1^β** | **^2^SE** | **^3^p** |
| rs964184 | *APOA1* | G | 0.056 | 0.044 | 0.205 | 0.031 | 0.047 | 0.513 |
| rs3764261 | *CETP* | A | 0.026 | 0.039 | 0.501 | -0.013 | 0.041 | 0.752 |
| rs646776 | *CELSR2-PSRC1-SORT1* | G | 0.054 | 0.040 | 0.172 | 0.034 | 0.042 | 0.420 |
| rs1412444 | *LIPA* | C | -0.011 | 0.036 | 0.764 | -0.002 | 0.039 | 0.950 |
| rs974819 | *PDGFD* | T | -0.065 | 0.037 | 0.078 | -0.010 | 0.040 | 0.798 |
| rs2954029 | *TRIB1* | T | 0.015 | 0.037 | 0.679 | -0.009 | 0.039 | 0.823 |

^1^β (Z score): within sib-pair coefficient of regression adjusted for age, sex, site (city) and location (rural/urban) and lipid traits ^2^SE: Standard Error. ^3^Loci were considered to be significant at α=0.0083 after false discovery rate for multiple testing

**Table S7: Within sib-pair association estimates for various** obesity traits

|  |  |  | **BMI** | | | **WHR** | | | **WC** | | | **% Body Fat** | | |
| --- | --- | --- | --- | --- | --- | --- | --- | --- | --- | --- | --- | --- | --- | --- |
| **SNP** | **Locus** | **Effect Allele** | **^1^β** | **^2^SE** | **^3^p** | **^1^β** | **^2^SE** | **^3^p** | **^1^β** | **^2^SE** | **^3^p** | **^1^β** | **^2^SE** | **^3^p** |
| rs964184 | *APOA1* | G | 0.030 | 0.039 | 0.438 | 0.049 | 0.040 | 0.221 | 0.080 | 0.041 | **0.050** | 0.007 | 0.032 | 0.817 |
| rs3764261 | *CETP* | A | -0.040 | 0.034 | 0.243 | 0.040 | 0.036 | 0.255 | 0.031 | 0.036 | 0.393 | -0.016 | 0.029 | 0.587 |
| rs646776 | *CELSR2-PSRC1-SORT1* | G | -0.019 | 0.035 | 0.593 | -0.022 | 0.036 | 0.537 | -0.008 | 0.037 | 0.830 | -0.005 | 0.029 | 0.873 |
| rs1412444 | *LIPA* | C | -0.011 | 0.032 | 0.733 | 0.050 | 0.033 | 0.130 | 0.024 | 0.034 | 0.482 | 0.011 | 0.027 | 0.689 |
| rs974819 | *PDGFD* | T | 0.045 | 0.032 | 0.161 | 0.018 | 0.034 | 0.593 | 0.023 | 0.035 | 0.505 | 0.016 | 0.027 | 0.562 |
| rs2954029 | *TRIB1* | T | -0.035 | 0.032 | 0.287 | -0.060 | 0.034 | 0.074 | -0.075 | 0.035 | **0.029** | -0.037 | 0.027 | 0.169 |

^1^β (Z score): within sib-pair coefficient of regression adjusted for age, sex, site (city) and location (rural/urban) and lipid traits. In case of estimation for BMI, WHR was also adjusted. ^2^SE: Standard Error. ^3^Loci were considered to be significant at α=0.0083 after false discovery rate for multiple testing. BMI: body mass index; WHR: waist-hip ratio; WC: waist circumference
